# Clear cell neuroendocrine tumor in the gallbladder diagnosed as a benign polyp preoperatively: a case report

**DOI:** 10.1186/s12957-020-02104-2

**Published:** 2021-01-02

**Authors:** Ryusuke Sumiya, Atsushi Shimizu, Takeshi Nagai, Hayato Ono, Keigo Kumazawa, Daisuke Endo, Takashi Oide, Nobuyoshi Aoyanagi

**Affiliations:** 1 Department of Surgery, Kohnodai Hospital, National Center for Global Health and Medicine, 1-7-1 Kohnodai, Ichikawa-shi, Chiba 272-8156 Japan; 2 Department of Pathology and Laboratory Medicine, Kohnodai Hospital, National Center for Global Health and Medicine, Ichikawa, Japan

**Keywords:** NET, Neuroendocrine neoplasm, Gallbladder, Endoscopic surgery, Case report

## Abstract

**Background:**

Gallbladder neuroendocrine neoplasm is a rare disease that is divided into neuroendocrine tumors (NETs) and neuroendocrine carcinomas (NECs). Clear cell NETs of the gallbladder are extremely rare. We report the case of a patient with polypoid clear cell NET G1 of the gallbladder who underwent laparoscopic cholecystectomy.

**Case presentation:**

A 10-mm pedunculated polyp in the gallbladder neck was detected on a follow-up abdominal ultrasound in a 60-year-old man with chronic hepatitis and hepatitis B without medication. Six months later, an abdominal ultrasound revealed that the tumor had enlarged to 12 mm in size. He was asymptomatic and had no abnormalities in other laboratory examinations, including the tumor markers, carcinoembryonic antigen and CA19-9. Abdominal ultrasound showed a 12-mm polyp in the neck of the gallbladder with perfusion and focal thickening of the gallbladder wall. A gallbladder stone was also seen in the fundus. An enhanced computed tomography scan and magnetic resonance imaging revealed a polypoid lesion and gallbladder stone located at the neck of the gallbladder and the fundus, respectively. Malignancy could not be excluded, and hence, a laparoscopic cholecystectomy was performed. Pathologically, a pedunculated polyp (14 × 11 × 15 mm) was observed in the neck of the gallbladder, and the polypoid lesion comprised nests or trabecular growths of clear NET cells in the lamina propria (ENETS: T1N0M0; AJCC: T1aN0M0). Immunohistochemical staining with synaptophysin, chromogranin A, and CD56 was confined to the tumor. The pathological diagnosis was clear cell NET G1 of the gallbladder. Although clear cell NET is often described as a distinct manifestation of von Hippel-Lindau disease (VHL), the patient had no past medical or family history of VHL. Until his one-and-a-half-year follow-up, the patient was doing well and without any signs of recurrence.

**Conclusion:**

We report an extremely rare case of gallbladder clear cell NET G1. When NET G1 is incidentally identified in a gallbladder surgical specimen, clinical information and pathological findings should be considered as references.

## Background

Neuroendocrine neoplasm (NEN), particularly gallbladder NEN, is a rare condition [1]. The World Health Organization 2018 classification divides NEN into two families. The well-differentiated family pertains to neuroendocrine tumor (NET), and the poorly differentiated family includes neuroendocrine carcinoma (NEC). NET is usually graded in three tiers, namely, G1, G2, and G3, which correspond to low-grade, intermediate-grade, and high-grade, respectively [2]. Among them, clear cell NET of the gallbladder is rare, and previous reports have revealed that several cases with clear cell NET are associated with von Hippel-Lindau (VHL) disease [3]. Although a patient with NET G1 has a relatively good prognosis, it is usually difficult to estimate the histological grade and to diagnose a gallbladder NEN during preoperative examination. Several cases of gallbladder NEN patients who underwent cholecystectomy because they were preoperatively diagnosed with a benign tumor have been reported [1]. Herein, we report a patient with polypoid clear cell NET G1 of the gallbladder who underwent laparoscopic cholecystectomy.

## Case presentation

**Fig. 1:**
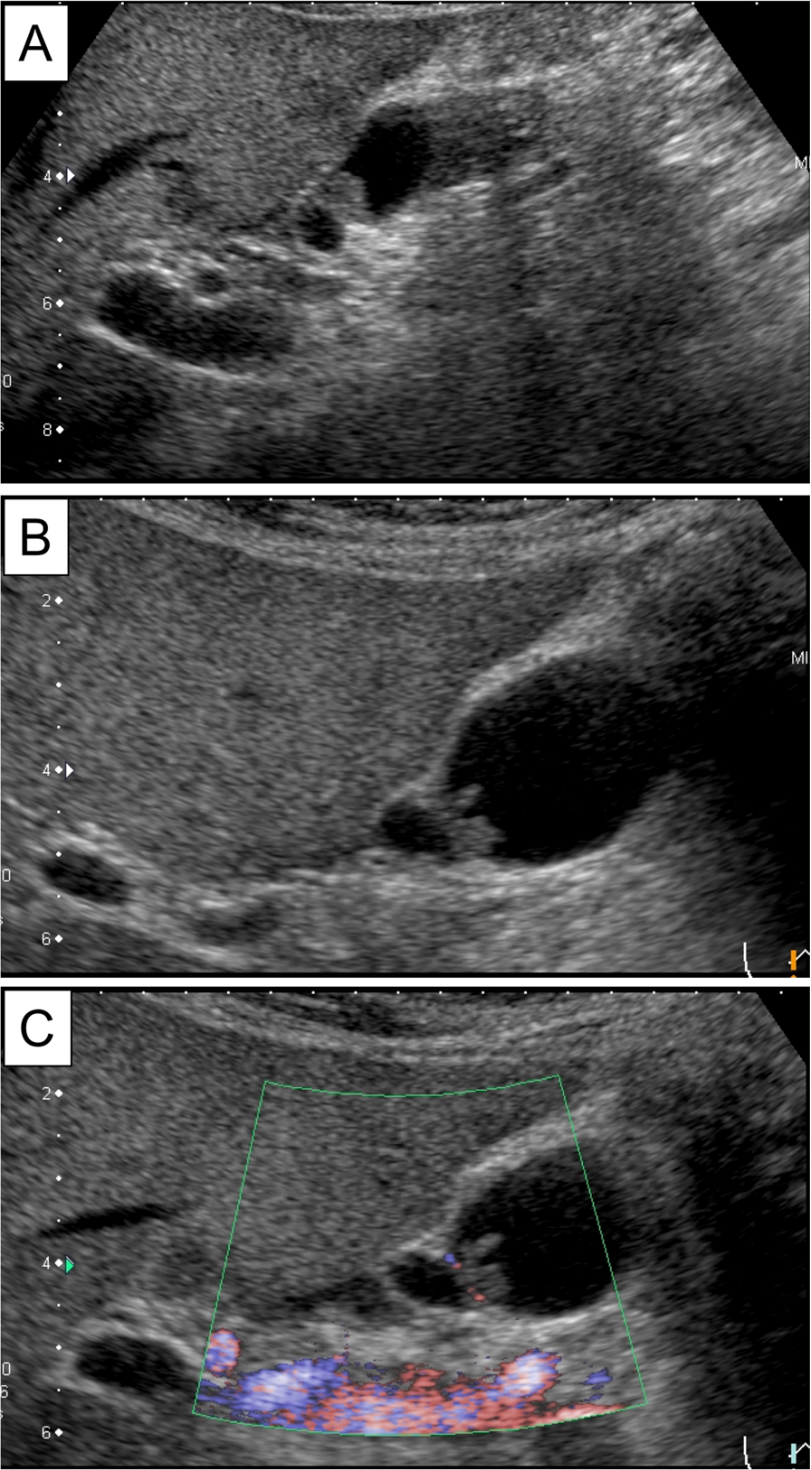
Representative images of the abdominal ultrasound. **a** A 10-mm polyp in the neck of the gallbladder was seen 6 months ago. **b** A 12-mm polyp in the neck of the gallbladder with perfusion and focal thickening of the gallbladder wall in the fundus. **c** Color Doppler imaging of the pedunculated polyp in the neck of the gallbladder

**Fig. 2:**
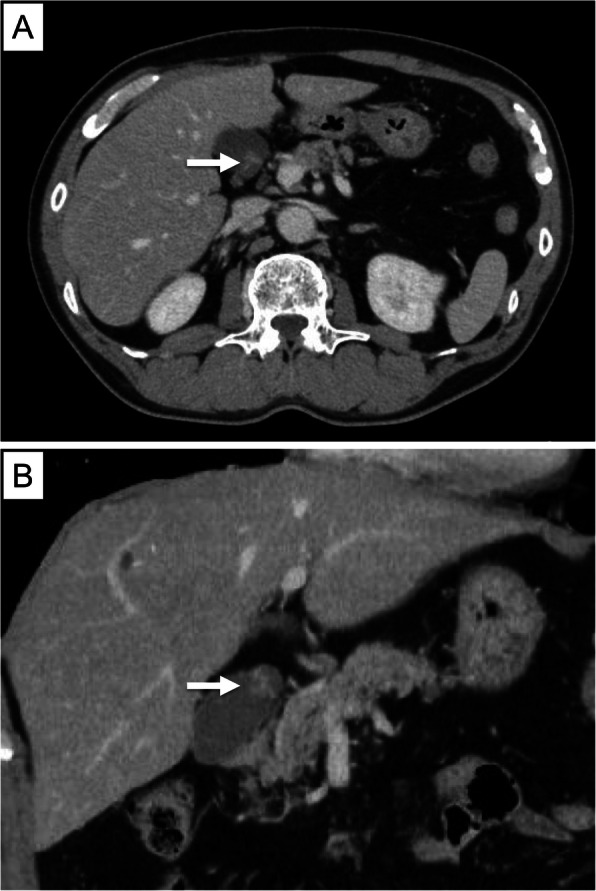
Representative images from the contrast-enhanced CT scan with portal venous phase. Arrow shows a pedunculated polypoid lesion in the neck of the gallbladder (**a**: axial image, **b**: coronal image)

**Fig. 3:**
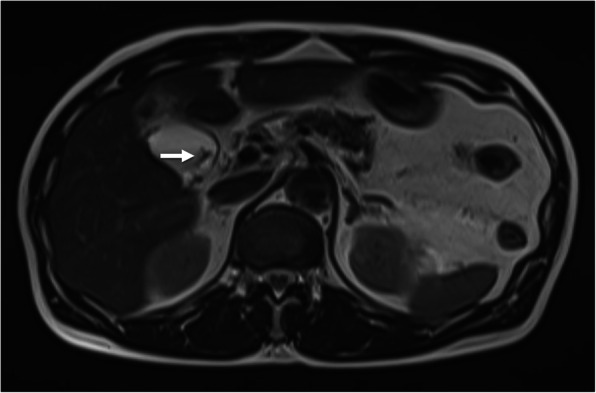
Representative image from the T2 axial MRI. A pedunculated polypoid lesion was observed in the neck of the gallbladder

**Fig. 4:**
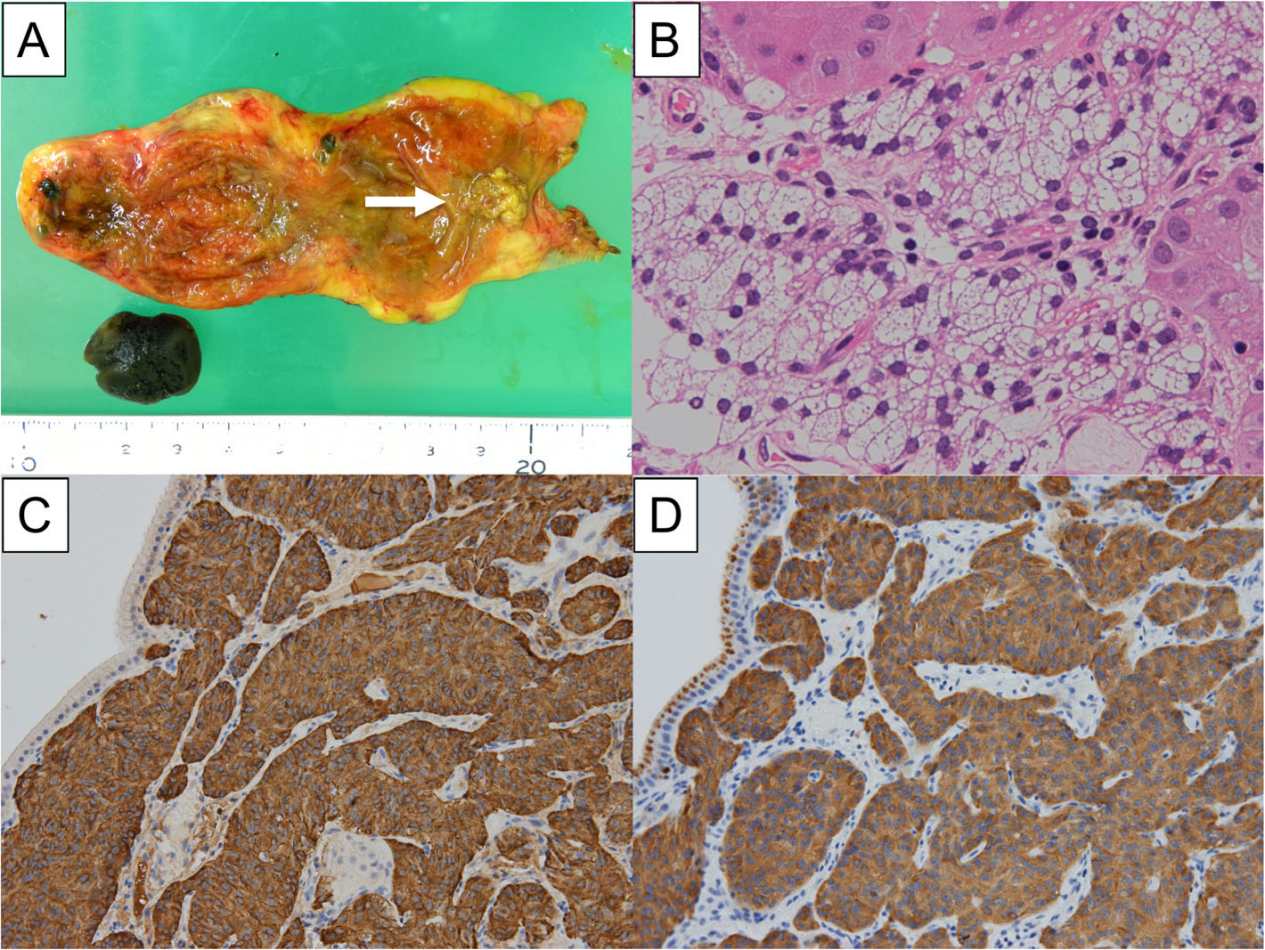
Representative images with pathological findings. **a** A pedunculated polyp (14 × 11 × 15 mm) was observed in the neck of the gallbladder (arrow), the gallbladder wall was thickened, and Rokitansky-Aschoff sinus (RAS) could be observed clustered in the wall. **b** The polypoid lesion comprised solid or trabecular nests of NET cells. These tumor cells had multivacuolated clear cytoplasm (hematoxylin and eosin staining, × 40 magnification). Immunohistochemically, the tumor cells were positive for synaptophysin (**c**) and chromogranin A (**d**)

A 10-mm pedunculated polyp in the gallbladder neck was detected on a follow-up abdominal ultrasound in a 60-year-old man with chronic hepatitis and hepatitis B without medication. A benign tumor or low-grade malignancy was suspected, and he initially did not wish to undergo surgical resection. Six months later, an abdominal ultrasound showed an increase in the polyp to 12 mm at the neck of the gallbladder with point-like perfusion, focal thickening of the gallbladder wall, and a gallbladder stone at the fundus (Fig. 1). Enhanced computed tomography (CT) scan (Fig. 2) and magnetic resonance imaging (MRI) (Fig. 3) revealed a polypoid lesion at the neck of the gallbladder, a distended gallbladder with a thickened and enhanced wall, and a gallbladder stone at the fundus. He was asymptomatic, and laboratory examinations revealed that the patient had a normal liver function and coagulation with hepatitis B virus DNA level of 3.2 log IU/ml; positive and negative serologic markers for hepatitis B envelope antibody and envelope antigen, respectively, and the tumor marker levels were within normal ranges [carcinoembryonic antigen, CEA (1.8 U/ml; normal range < 5.0 U/ml) and CA19-9 (9.9 U/ml; normal range < 37 U/ml)]. Although the patient was diagnosed with a benign gallbladder polyp, malignancy could not be completely excluded. Therefore, a laparoscopic cholecystectomy was performed. Intraoperatively, only a slight thickening of the gallbladder wall was detected. Macroscopically, there was a pedunculated yellowish polyp (14 × 11 × 15 mm) at the neck of the gallbladder. The gallbladder wall was thickened, and the Rokitansky-Aschoff sinus (RAS) was clustered at the wall (Fig. 4a).

Histopathological examination revealed solid or trabecular nests of tumor cells in the polypoid lesion. These tumor cells were uniformly monotonous with a small round nucleus and multivacuolated clear cytoplasm (Fig. 4b), and the cytoplasm was negative for periodic acid-Schiff reaction. Necrosis and mitosis were not observed. The surface of the polyp was composed of a biliary epithelium without atypia. The neoplastic lesion was restricted to the lamina propria, and neither vascular nor lymphatic invasion was present (ENETS: T1N0M0; AJCC: T1aN0M0). Chronic cholecystitis with RAS was observed in the surrounding gallbladder tissue. Immunohistochemically, the tumor cells were positive for synaptophysin, chromogranin A, and CD56, indicating neuroendocrine differentiation (Fig. 4c, d). The expression of Ki-67 showed a labeling index of < 2%. Hence, the tumor was diagnosed as clear cell NET G1 of the gallbladder. No additional treatment was administered because R0 resection was performed using cholecystectomy, and there was no evidence of invasion or metastasis to other organs. Although clear cell NET may appear as a part of VHL disease, the patient had no clinical findings and family history of VHL. The patient is doing well without any signs of recurrence until the one-and-a-half-year follow-up period.

## Discussion and conclusion

NEN is a rare disease, and its prevalence is approximately 5.25 per 100,000 [1]. NEN has been described in the gastrointestinal tract, lungs, trachea, and thyroid. However, NEN rarely occurs in the gallbladder, accounting for only 0.04–0.5% of all NEN cases [4, 5]. In particular, gallbladder NET with clear cell is rare, with only four reported cases (Table 1) [3, 6–8]. Since little is known about the clinicopathological features of clear cell NEN in the gallbladder, we present this rare case of gallbladder NEN with clear cell in this report.

**Table 1:** Reported cases of clear cell NET G1 in the gallbladder

No.	Age	Sex	Location	Form	Preoperative diagnosis	Size (mm)	Operation	Invasion depth	Metastasis
1 [3]	38	M	Neck of gallbladder	Pedunculated	Metastasis of renal cancer	14	Lap cholecystectomy	Full thickness	None
2 [6]	64	M	Neck of gallbladder	Pedunculated	Cholesterol polyp	8	Lap cholecystectomy	Subserosal layer	None
3 [7]	71	M	Fundus of gallbladder	Pedunculated	Polyp	9	Lap cholecystectomy	Lamina propria	None
4 [8]	65	M	Neck of gallbladder	Non-pedunculated	RAS	8	Lap cholecystectomy + radical second resection + lymphadenectomy	Muscular layer	Lymph node
5^a^	70	M	Neck of gallbladder	Pedunculated	Polyp	15	Lap cholecystectomy	Lamina propria	None

^a^Present case

The mechanisms of occurrence of gallbladder NEN have not been explained because the normal gallbladder mucosa has no neuroendocrine cell. Previous research reported that the proportion of neuroendocrine cells increases during chronic inflammation in the affected metaplastic epithelium, which may result in NEN. This explains why patients with gallbladder NEN often have gallbladder stones and cholecystitis [9]. A previous study revealed that 65% of gallbladder NETs frequently arise from the neck of the gallbladder, while 35% occur in the body or fundus. Although 61.5% of gallbladder NETs in the neck are pedunculated, 38.5% of gallbladder NETs in the neck and all gallbladder NETs in the body and fundus are not pedunculated [10]. Almost all gallbladder NEN patients are asymptomatic, and the proportion of hormone-producing gallbladder NENs is unknown because of the small number of reported cases [11]. Regarding clear cell gallbladder NET, most cases were in the neck of the gallbladder and pedunculated, and all cases were surgically resected by laparoscopic cholecystectomy. Only one case underwent a radical second resection and lymphadenectomy because the patient had a cystic lymph node metastasis. However, since cystic lymph node metastases were absent in the other cases and present case, radical resection was not necessary.

Due to the lack of specific findings on the CT scan or MRI, it is difficult to diagnose gallbladder NEN preoperatively. Gallbladder NEN is often misdiagnosed as a benign tumor, i.e., cholesterol polyp or adenoma prior to surgery. If the diagnosis of gallbladder NEN was made preoperatively, we should have considered operative methods such as local excision, radical resection, and lymphadenectomy. Previous research recommends that local excision without lymphadenectomy should be carried out for NET G1 tumors less than 2 cm in the stomach, duodenum, appendix, and rectum. Lymphadenectomy is recommended for other gastroenteropancreatic NENs in the jejunum, ileum, colon, and pancreas [9, 11]. Although the risk of metastasis depends on the tumor size in several gastroenteropancreatic NENs, the risk factors for metastasis of gallbladder NEN have not been clarified because only a small number of cases have been reported [11]. Previous NET research reported that the frequency of metastases was two of seven cases (28.6%) for tumors less than 1 cm, while all five cases with tumors measuring 3 cm or more had metastases. This research indicated that tumor size is a possible risk factor of metastases for gallbladder NEN [12]. Frequent sites of metastatic lesions were the liver (91.7%), lungs (33.3%), lymph nodes (33.3%), bone (25%), and adrenal glands (25%) [8–12]. Only one case of gallbladder NET G1 less than 1 cm had lymph node metastasis [8]. MRI is a useful tool in staging gallbladder tumors, and previous research revealed the high sensitivity rates for lymph node invasion on MRI (92%) [13]. Therefore, we elucidated the tumor size and lymph node metastasis on MRI preoperatively. Postoperatively, if NET G1 was incidentally identified in the gallbladder of a surgical specimen, reports suggested that a detailed pathological examination of the cystic duct node should be performed [8]. If lymph node metastasis is positive, a radical second resection with regional lymphadenectomy should be considered. In this case, a small tumor size (1.5 cm) and histological type (NET G1) were associated with a good prognosis without metastasis. In addition, preoperative MRI showed no evidence of lymph node metastasis.

Generally, the cytoplasm of NEN cells is eosinophilic to amphophilic. However, few cases have a clear cytoplasm, which are described as clear cell NEN. A previous report showed that clear cell NEN of the pancreas is a clinical presentation of VHL [14]. Only four cases of clear cell gallbladder NEN have been described in previous literature, and one case was associated with VHL [8]. In the other three cases, i.e., non-VHL-related cases, prior reports indicated that chronic cholecystitis and gallbladder stones may be associated with clear cell NET G1, likewise the pathogenesis of canonical gallbladder NEN [6–8]. This patient had no clinical feature and family history of VHL disease.

In conclusion, while gallbladder NEN is rare and clear cell NET has been reported as a clinical presentation of VHL, this case of gallbladder clear cell NET G1 is particularly notable due to the absence of VHL. Further, we showed that G1 NETs less than 2 cm without lymph nodes and distant metastases can be treated with laparoscopic cholecystectomy and local excision. However, further research is necessary to evaluate imaging findings prior to surgery, to determine the surgical procedure, and to identify the potential prognostic factors. When NET G1 is incidentally identified in a gallbladder surgical specimen, clinical information and pathological findings should be considered as references.

## Data Availability

Not applicable.

## Abbreviations

NEN: Neuroendocrine neoplasm
NET: Neuroendocrine tumor
NEC: Neuroendocrine carcinoma
VHL: von Hippel-Lindau
CT: Computed tomography
MRI: Magnetic resonance imaging
